# Patterns of cross‐resistance and collateral sensitivity between clinical antibiotics and natural antimicrobials

**DOI:** 10.1111/eva.12762

**Published:** 2019-01-28

**Authors:** Abigail Colclough, Jukka Corander, Samuel K. Sheppard, Sion C. Bayliss, Michiel Vos

**Affiliations:** ^1^ European Centre for Environment and Human Health University of Exeter Medical School Cornwall UK; ^2^ Institute of Microbiology and Infection University of Birmingham Birmingham UK; ^3^ Department of Biostatistics University of Oslo Oslo Norway; ^4^ Department of Mathematics and Statistics University of Helsinki Helsinki Finland; ^5^ The Milner Centre for Evolution, Department of Biology & Biochemistry University of Bath Bath UK

**Keywords:** antibiotic resistance, Antimicrobials, collateral sensitivity, cross‐resistance, seaweeds, *Staphylococcus aureus*

## Abstract

Bacteria interact with a multitude of other organisms, many of which produce antimicrobials. Selection for resistance to these antimicrobials has the potential to result in resistance to clinical antibiotics when active compounds target the same bacterial pathways. The possibility of such cross‐resistance between natural antimicrobials and antibiotics has to our knowledge received very little attention. The antimicrobial activity of extracts from seaweeds, known to be prolific producers of antimicrobials, is here tested against *Staphylococcus aureus* isolates with varied clinical antibiotic resistance profiles. An overall effect consistent with cross‐resistance is demonstrated, with multidrug‐resistant *S. aureus* strains being on average more resistant to seaweed extracts. This pattern could potentially indicate that evolution of resistance to antimicrobials in the natural environment could lead to resistance against clinical antibiotics. However, patterns of antimicrobial activity of individual seaweed extracts vary considerably and include collateral sensitivity, where increased resistance to a particular antibiotic is associated with decreased resistance to a particular seaweed extract. Our correlation‐based methods allow the identification of antimicrobial extracts bearing most promise for downstream active compound identification and pharmacological testing.

## 1 INTRODUCTION

Bacterial cross‐resistance can be defined as resistance to multiple distinct antimicrobial agents conferred by a single molecular mechanism. It occurs when antimicrobials share a route of access to the cytoplasm, bind the same target or are involved in the same pathway leading to the inhibition of growth or cell death (Baker‐Austin, Wright, Stepanauskas, & McArthur, 2006). This phenomenon is best described in the context of shared resistance between different clinical antibiotic classes (Sanders, Sanders, Goering, & Werner, 1984), between antibiotics and disinfectants, biocides or solvents (Chapman, 2003; Chuanchuen et al., 2001; Fernandes, Ferreira, & Cabral, 2003) and between antibiotics and heavy metals (Baker‐Austin et al., 2006). An example of cross‐resistance is the efflux system AcrAB–TolC which confers resistance to multiple classes of antimicrobials but also to metals, dyes and detergents (Anes, McCusker, Fanning, & Martins, 2015). It is increasingly realized that bacterial exposure to anthropogenic antimicrobials in wastewater, agricultural settings or the built environment, has the potential to co‐select for resistance to clinical antibiotics and significantly contribute to the rise of antibiotic‐resistant pathogens (Wellington et al., 2013). However, the potential of the natural environment to co‐select for antibiotic resistance has received little attention (Allen et al., 2010). As the genetic mechanisms conferring antibiotic resistance are ancient and many of the selective forces that can promote the spread of these mechanisms are potentially nonanthropogenic, this is of concern.

One potential avenue of selection for antibiotic resistance in the natural environment is selection for resistance driven by antimicrobial‐producing organisms. Virtually all organisms, from bacteria to humans, produce antimicrobial compounds (Raaijmakers, Vlami, & De Souza, 2002; Zasloff, 2002). The ubiquity of interactions between bacteria and antimicrobial producers offers great potential for the molecular diversification of bacterial resistance mechanisms, a subset of which might also confer resistance to clinical antibiotics. To our knowledge, there have been no previous investigations into the level of cross‐resistance of natural antimicrobials with clinical antibiotics. This lack of data is problematic as environmental reservoirs of resistant bacteria or resistance genes could make their way back into the clinic or community and cause hard‐to‐treat infections.

The opposite effect of cross‐resistance is collateral sensitivity, where pleiotropic effects cause resistance to natural antimicrobials to be negatively correlated with resistance to antibiotics (Pál, Papp, & Lázár, 2015). One example is the evolution of aminoglycoside resistance through mutations resulting in a reduction in the proton‐motive force, leading to a diminished activity of efflux pumps involved in resistance to a range of other antibiotics classes (Lázár et al., 2013). Such trade‐offs between two antimicrobials can be exploited for clinical use in the form of combination therapy (Pál et al., 2015). Optimizing discovery strategies for novel antimicrobials that display collateral sensitivity with clinical antibiotics could be a promising strategy to combat multidrug‐resistant bacterial pathogens.

We here use pathogenic *Staphylococcus aureus* isolates and seaweeds as a model for cross‐resistance and collateral sensitivity between clinical antibiotics and natural antimicrobials. *S. aureus* is an opportunistic pathogen, and strains resistant to multiple antibiotics (including methicillin [MRSA] [12]) are causing increased mortality and costs of care (De Kraker, Davey, & Grundmann, 2011; Macedo‐Viñas et al., 2013; Rubio‐Terrés, Garau, Grau, & Martinez‐Martinez, 2010). Seaweeds (or “macroalgae”) form a diverse and abundant component of coastal ecosystems. Seaweeds lack cell‐based immune responses but are continually exposed to a large variety of potentially harmful microorganisms present in seawater. It has therefore been hypothesized that they commonly exhibit antimicrobial activity to prevent fouling and disease (Goecke, Labes, Wiese, & Imhoff, 2010; Plouguerne, Hellio, Deslandes, Véron, & Stiger‐Pouvreau, 2008; Weinberger, 2007). A large number of studies have demonstrated that extracts of many seaweed species are able to kill or inhibit Gram‐positive and Gram‐negative bacteria, including nonmarine human pathogens such as *S. aureus* (e.g., Horikawa, Noro, & Kamei, 1999; Pierre et al., 2011).

We pair a large set of diverse *S. aureus* strains isolated from human infections with extensively characterized clinical antibiotic resistance spectra to a collection of seaweed extracts. We use quantitative measures of susceptibility, allowing detailed correlation analyses on the efficacy of antimicrobial extracts. We first test whether multidrug‐resistant bacterial display greater on average levels of resistance to seaweed extracts. We next analyse patterns indicative of cross‐resistance and collateral sensitivity between individual extracts and clinical antibiotics. Our results both shed light on the potential of macroalgae to select for antibiotic resistance in the bacteria that settle on them and have the potential to inform strategies of natural product discovery.

## 2 MATERIAL AND METHODS

### 2.1 Seaweed extracts

Seaweeds were collected along the southwest coast of Cornwall (UK). Intertidal species were collected at low tide, with subtidal species retrieved by scuba diving. Seaweeds were carefully inspected, and epiphytes and necrotic areas were removed, followed by rinsing with ddH_2_0. Washed samples were sealed in individual bags and stored at −20°C until extraction. Samples were lyophilized using a freeze drier (Scanvac, Labogene, Lynge, Denmark) and ground using a household spice grinder (James Martin ZX809X). The resulting powder was mixed with 60% methanol (5 g in 50 ml) and incubated for 2 hr at 40°C at 100 rpm. After extraction, samples were centrifuged at 1,000 *g* for 15 min, after which the supernatant was evaporated and resuspended to a final volume of 5 ml in a fume hood. Concentrated extract was aliquoted and stored at −80°C until further use.

### 2.2 Bacterial strains

Twenty‐eight pathogenic *Staphylococcus aureus *isolates were obtained from the Royal Cornwall Hospital in Truro (UK). VITEK 2 AST (bioMérieux, Marcy‐l’Étoile, France) data detailing antibiotic susceptibility were provided for each strain after removing patient data. Individual colonies were picked and cultured in 5 ml LB broth (37°C, 100 rpm) and stored as 20% glycerol freezer stocks at −80°C.

### 2.3 Kirby‐Bauer disc diffusion assay

A Kirby‐Bauer disc diffusion assay (Bauer, Perry, & Kirby, 1959) was performed using overnight cultures (18 hr) diluted in broth to a turbidity equivalent to a McFarland standard (Andrews, 2013) of 0.5 at 625 nm as measured by spectrophotometry (Bibby Scientific Limited, Staffordshire, UK). Four hundred micro litre of this dilution was mixed with 30 ml of sterile Mueller‐Hinton agar (Oxoid, Basingstoke, UK) and poured into square plates (Gosselin, Borre, France). Whatman AA assay discs (Whatman International Limited, Maidstone, UK), soaked in seaweed extract for 24 hr, were dried in a laminar flow hood for 15 min. Positive control (imipenem, 4 mg/L) and negative control (60% methanol) discs were soaked and dried in the same way. Dry discs were placed on the agar using sterile tweezers (14 seaweed extract discs and two controls per plate, all combinations were plated in duplicate). Plates were incubated at 37°C. After 18 hr, zones of inhibition (areas with no visible bacterial growth) were measured for each disc. Inhibition zone sizes were recorded as total diameter minus size of the diffusion disc (5 mm).

### 2.4 Minimum inhibitory concentration

Seaweed extracts (200 µl) were evaporated overnight in the first column of a 96‐well plate (Starlab Limited, Milton Keynes, UK). A twofold dilution range of each extract (200 µl volume) was made in nine columns in LB broth, with the remaining two columns used as positive and negative controls. Diluted bacterial inoculum was added to a final concentration of 5 × 10^5^ CFU/ml. 10 μl alamar blue dye (Thermo Fisher Scientific Incorporated, Waltham, USA) was added as an indicator of bacterial respiration (growth). Plates were briefly agitated and incubated at 37°C for 18 hr. Antimicrobial susceptibility to Tetracycline, Oxacillin, Cefotaxime, Gentamicin, Rifampicin and Erythromycin (Sigma‐Aldrich, Steinheim, Germany) was tested for a subset of strains. Stock solutions (10 mg/ml) were prepared in ddH_2_O and filter sterilized. A broth microdilution assay was performed in a 96‐well plate as described above, using antibiotic concentrations from 0.1 to 100 mg/L.

### 2.5 Genome sequencing, bioinformatics and phylogenetic analyses

A phenol:chloroform:isoamyl alcohol DNA isolation protocol modified from Sambrook and Russel (Sambrook, 2006) was used to obtain genomic DNA. DNA quality was visually assessed on a 1% agarose gel, and DNA quality was measured using Qubit fluorometer (Life Technologies). DNA was dissolved in Elution Buffer (10 mM TRIS, pH8) and sent on dry ice to the University of Exeter Sequencing Facility. Sequencing libraries were run on a Hiseq 2,500 in rapid run mode (250 base pair paired‐end reads) yielding between 1.3 and 6 million reads per sample. Sequencing data were trimmed to remove sequencing adaptors and low‐quality terminal ends (<Q20) using fastq‐mcf v1.1.2‐537 (Aronesty, 2011). Reads were assembled using SPAdes 3.11.0 (Bankevich et al., 2012) and assessed using QUAST (Gurevich, Saveliev, Vyahhi, & Tesler, 2013). Small contigs (<500 base pairs) were removed. Short reads were mapped to the reference genome H0 5096 0412 (EMRSA15) using SMALT on default settings and a mean insert size of 300 bp (http://www.sanger.ac.uk/resources/software/smalt/). Reads containing insertions or deletions (indels) were realigned using the Genome Analysis Toolkit's IndelRealigner (https://www.ncbi.nlm.nih.gov/pmc/articles/PMC2928508/). Single nucleotide polymorphisms (SNPs) were called using SAMtools 0.1.18 (H. Li et al., 2009). Variants were filtered using in‐house scripts to include only SNPs with >4x read depth per base (>2 per strand), >75% support for an alternative variant, mapping quality >30 and a site allele frequency of the alternative allele of >0.95. Samples were sequence typed using ARIBA from the pubMLST databases accessed on 26 January 2017 (Hunt et al., 2017). A maximum‐likelihood phylogenetic tree was reconstructed for core genomes using RAxML (Stamatakis, 2014). Consensus sequences generated by the variant calling pipeline were passed to RAxML under a general time‐reversible (GTR) model of nucleotide substitution with a GAMMA rate of site heterogeneity. A rapid bootstrap analysis (100 bootstraps) and search for best‐scoring ML tree were performed in a single program run.

### 2.6 Statistical analyses

Test, correlation covariance and ANOVA were performed in R (version 0.98.1103, R Core Team, 2013; Team, 2015). Dedicated packages corrplot and survival were used for survival analysis (Therneau & Lumley, 2015; Wei & Wei, 2015). The packages gplots (Warnes et al., 2013) and ggplot2 (Wickham, 2009) and lattice (Sarkar & Sarkar, 2015) were used for graphics. To categorize the extracts into those with nearly constant effect and others with a more variable effect, the k‐means algorithm (Bishop, 2006) was used on inhibition zone standard deviations with two classes and the default settings in MATLAB (Release, 2012). To maximize power while accounting for multiple hypothesis testing, a standard permutation test (Gao, Becker, Becker, Starmer, & Province, 2010) was used to identify individual extracts in the more variable class with significant association between the level of clinical (VITEK) resistance and the halo sizes. Correlations between the clinical resistance and the average inhibition zone size from replicate measurements for each extract were calculated and tested for significance using 100,000 random permutations of clinical resistance values. Trees and figures were visualized using the R package ggtree (Yu, Smith, Zhu, Guan, & Lam, 2017).

## 3 RESULTS

### 3.1 Resistance to seaweed extracts as a function of multidrug resistance

A total of 48 species of macroalgae were collected from intertidal and shallow subtidal waters in Cornwall, UK (Table S1) and processed into methanolic extracts of standardized dry weight concentration. Extracts were used in a disc diffusion assay (Figure S1) to challenge 28 clinical *S. aureus* strains, each with a unique clinical antibiotic susceptibility profile (Table S2). Beta‐lactam resistance was observed at high frequency within the collection: 26 isolates were resistant to benzylpenicillin. Fourteen isolates were methicillin resistant, and all of these MRSA isolates were also resistant to oxacillin and broad spectrum cefoxitin. Intermittent resistance to fusidic acid, clindamycin, erythromycin and tetracycline was observed (Table S2).

**Figure 1:**
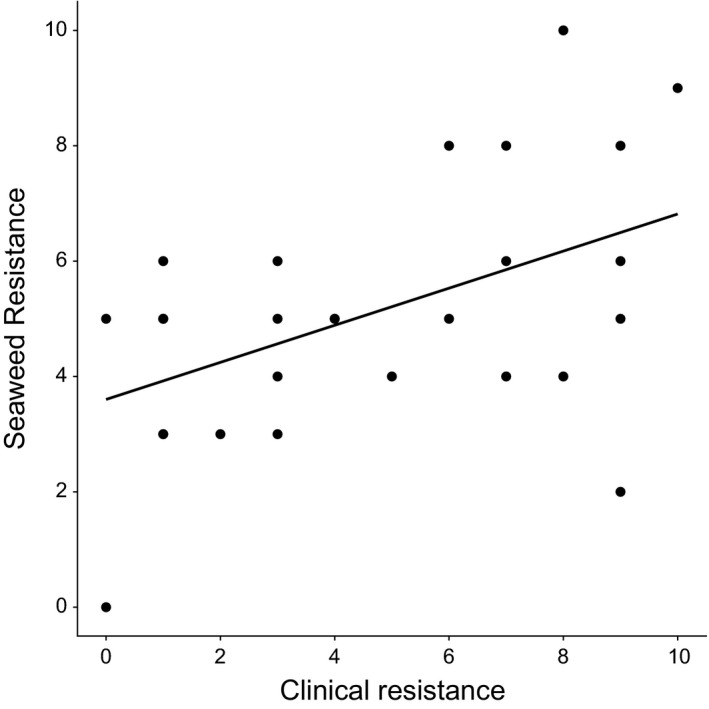
Correlation between clinical resistance (sum of 22 antibiotics assayed using VITEK technology) and seaweed resistance (sum of 27 methanolic extracts) for 28 *S. aureus* isolates (*R* ^2^ = 0.21, *p* < 0.01).

**Figure 2:**
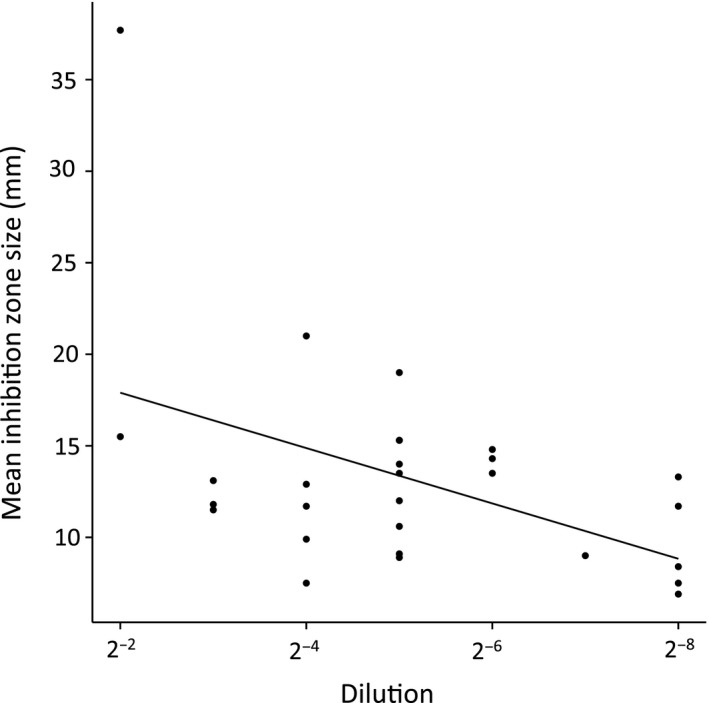
Correlation between dilution factor and inhibition zone size for 27 extracts assayed on strain SA2934 (*R* ^2^ = 0.21, *p* < 0.01). Dilution factor is inversely proportional to Minimal Inhibitory Concentration (MIC).

**Figure 3:**
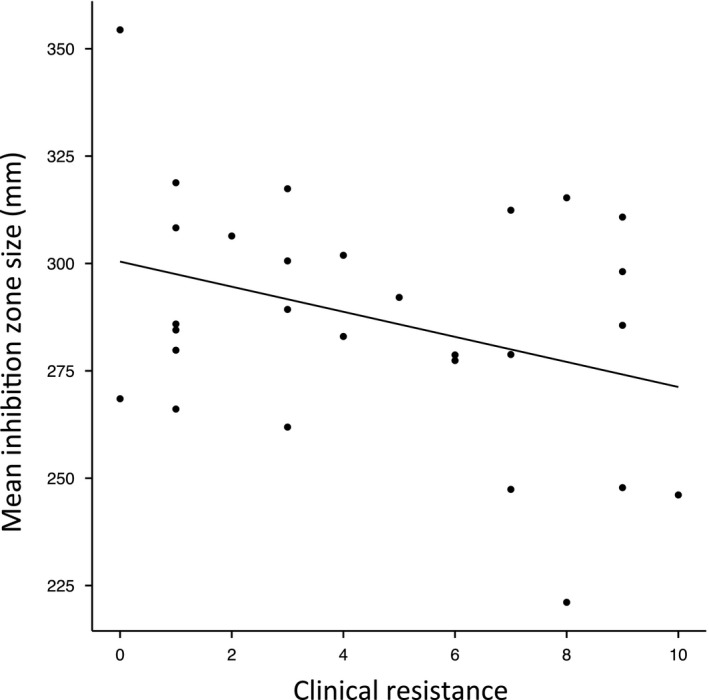
Correlation between resistance against clinical antibiotics (assayed using VITEK technology) and seaweed resistance as quantified by the sum of inhibition zone sizes of the 27 seaweed extracts able to inhibit all 28 *S. aureus* isolates (*R* ^2^ = 0.08, *p* < 0.07).

27/48 (56%) of extracts showed activity against at least one *S. aureus* strain, as indicated by a clear zone of growth inhibition. A total of 17 extracts inhibited all 28 bacterial strains, whereas ten extracts showed inhibition against a subset of strains (Table S1). Plotting the number of clinical antibiotics against the number of seaweed extracts each strain was resistant to reveals a significant positive correlation (Figure 1; *R* ^2^ = 0.21, *p* < 0.01), indicating that on average, antimicrobials isolated from natural sources are least effective against the most problematic multidrug‐resistant strains. In addition to scoring presence or absence of inhibition zones, the effect of individual seaweed extracts was analysed by quantifying inhibition zone sizes. Inhibition zone size is significantly negatively correlated with the Minimum Inhibitory Concentration (minimum inhibitory concentration [MIC], the lowest concentration at which bacterial growth is inhibited) based on pairing 27 active extracts with the strain most sensitive to clinical antibiotics (*R* ^2^ = 0.21, *p* < 0.01; strain SA2934, Figure 2). Strains resistant to a greater number of clinical antibiotics showed a tendency to display a smaller total inhibition zone size (sum of inhibition zone sizes of the 27 seaweed extracts) (*R* ^2^ = 0.08, *p* = 0.07; Figure 3), consistent with the data in Figure 1.

### 3.2 Resistance to individual seaweed extracts as a function of multidrug resistance

**Figure 4:**
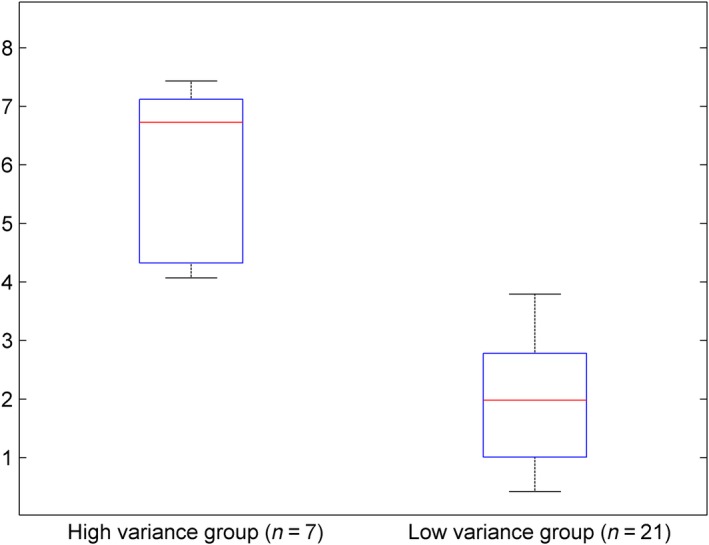
Box‐plots of inhibition zone size standard deviations for a high and low variance seaweed antimicrobial activity group created by the k‐means algorithm with default settings

In order to identify individual extracts that showed a significantly positive or negative relationship with overall clinical resistance, a k‐means algorithm was used on inhibition zone size standard deviations to divide extract in a high and a low variance class. The majority of extracts (21) show low variation in activity across the panel (Figure 4). Next, a permutation test was used on the seven high variance species (*Chaetomorpha melagonium*, *Ulva lactuca, Cladophora rupestris*, *Ceramium rubrum*, *Spyridia griffithsiana*, *Corallina officinalis* and *Plumaria plumosa*) to test whether their activity was significantly correlated with clinical resistance as determined by VITEK. Three extracts displayed moderately strong negative association with clinical resistance, with halo size decreasing with increasing clinical resistance, of which two were highly significant (*C. rupestris*: *r* = −0.487, *p* = 0.00423; *C. rubrum*: *r* = −0.463, *p* = 0.00681) and one borderline nonsignificant (*U. lactuca*: *r* = −0.306, *p* = 0.0567). No extracts were found to have a significantly positive association between inhibition size and clinical resistance.

### 3.3 Cross‐resistance and collateral sensitivity patterns between individual seaweed extracts and antibiotics

**Figure 5:**
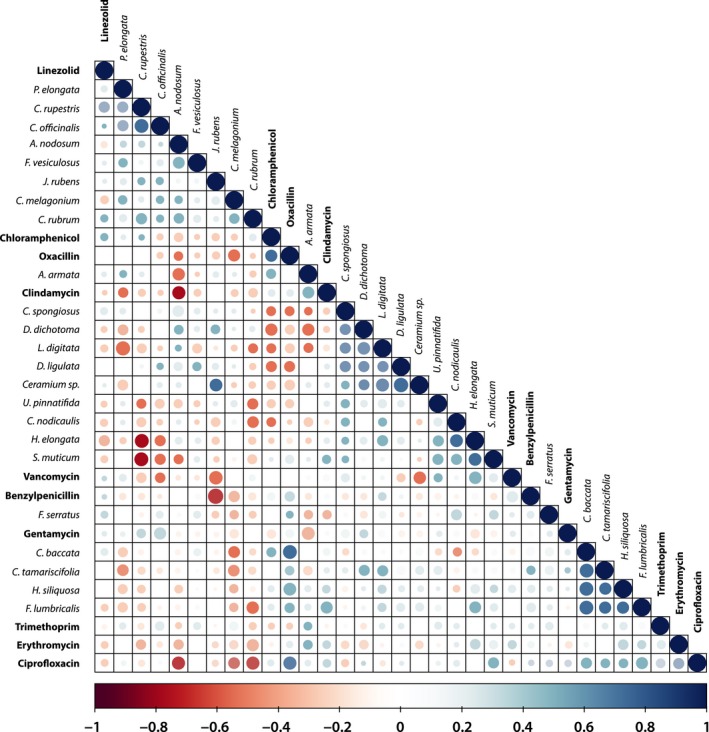
Pearson correlation coefficients between seaweed extract inhibition zone sizes and clinical antibiotic MICs assayed using VITEK technology generated on a test panel of 28 *S. aureus* isolates. Colour‐coded values range from −1 = perfect negative correlation (red) to 1 = perfect positive correlation (blue); the size of the data points co‐varies with colour intensity

A more detailed analysis based on extract inhibition zone sizes for 27 seaweed extracts and MICs for 14 different antibiotics was performed using Pearson correlation coefficients (Figure 5). This analysis shows that the activities of some seaweeds across the *S. aureus* panel are similar, suggesting that they produce similar antimicrobial compounds. In some cases, seaweed extracts show activity patterns similar to those of antibiotics, for example, *Cystoseira baccata, Cystoseira tamariscifolia* and oxacillin (Figure 5). However, the opposite pattern of cross‐resistance, where the activity of antibiotics is negatively correlated with the activity of seaweed extracts, also occurs, for example, benzylpenicillin and *Jania rubens* or ciprofloxacin and *C. melagonium*, *C. rubrum* and *Ascophyllum nodosum *(Figure 5).

### 3.4 Antimicrobial activity as a function of seaweed relatedness

**Figure 6:**
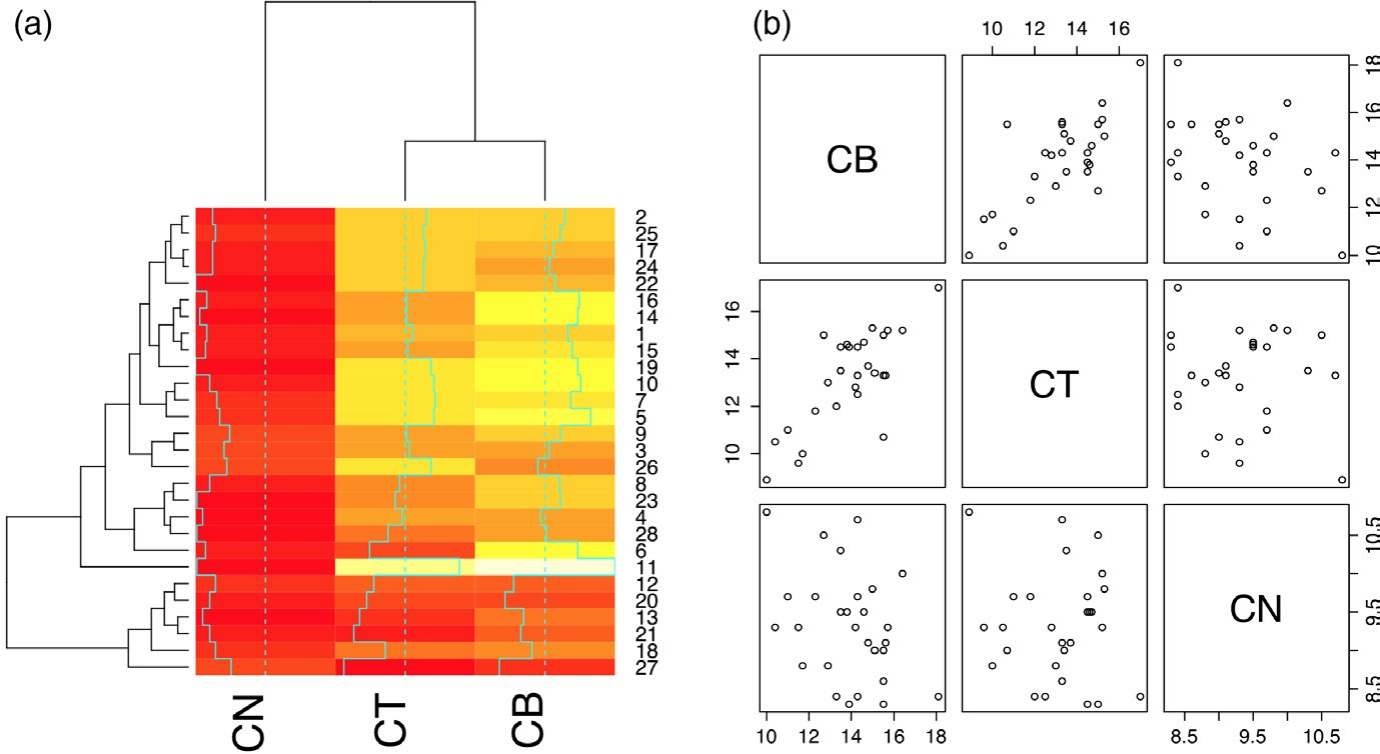
Differences in anti‐*S. aureus* activity between three *Cystoseira* species: *C. tamariscifolia* (CT), *C. baccata* (CB) and *C. nodicaulis* (CN) based on the average of two independent replicates. (a) Heatmap showing inhibition zone size (yellow: small, red: large) for each of the three extracts on 28 *S. aureus* isolates clearly demarcates the CN extract as having differential activity. (b) A correlation matrix plotting inhibition zone sizes for pairs of extracts on the *S. aureus* panel

To test whether closely related seaweeds species had similar antimicrobial activity, we focused on the only genus represented by three species: *Cystoseira* (*C. tamariscifolia*, *C. baccata* and *C. nodicaulis*). Two of the three species were found to have highly similar effects on the panel of 28 *S. aureus* strains whereas a third species had a noticeably different overall effect, as highlighted by a heatmap (Figure 6a). Differential inhibition could in theory be due the production of different active compounds (i.e., qualitative differences) or due to differences in concentration of compounds (i.e., quantitative differences) between extracts. By plotting inhibition zone sizes on all 28 *S. aureus* strains for the three different pairwise extract combinations (Figure 6b), it is possible to distinguish between these two scenarios. A specific extract can be expected to show the same qualitative effect on the panel of bacteria regardless of its concentration (i.e., a more diluted extract will show proportionally smaller inhibition zones). That there is no significant correlation between the outlier species *C. nodicaulis* and the two other species indicates that its extract is qualitatively different (Figure 6b).

A previous study demonstrated that of these three *Cystoseira* species, *C. tamariscifolia* was the out‐group on the basis of the ITS2 sequence and two physicochemical methods (Jégou, Culioli, Kervarec, Simon, & Stiger‐Pouvreau, 2010). A disc diffusion assay where the three *Cystoseira* extracts were tested against three Gram‐negative species confirmed the out‐group position of *C. tamariscifolia* (Figure S2). Together, these findings demonstrate that genetic divergence, metabolomic divergence and antagonistic activity on other bacterial types are not reliable indicators of antimicrobial activity. Differences in antagonistic activity were observed for two *Fucus* species, two *Ulva* species and two *Ceramium* species, further supporting the observation that antimicrobial activity can vary within seaweed genera (data not shown).

### 3.5 Genomic context of seaweed extract resistance

**Figure 7:**
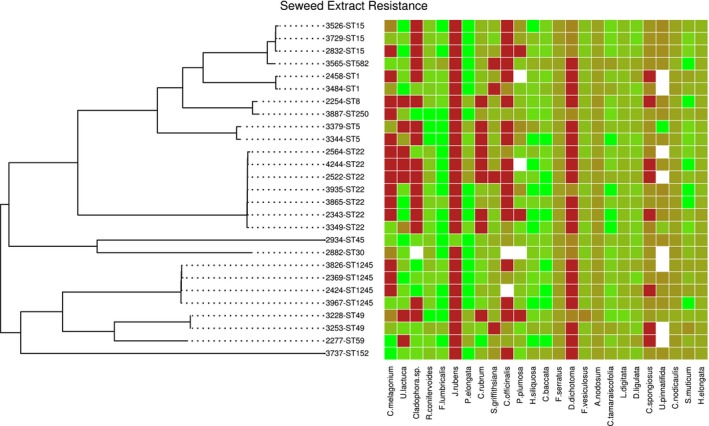
A maximum‐likelihood phylogenetic tree based on whole genome sequence data of 27 *S. aureus* genomes used in this study mapped to EMRSA15 reference genome HO 5,096 0,412 (not included in the tree) (2,832,299 bp). The panel on the right indicates susceptibility to 25 seaweed extracts, quantified by zone of inhibition (red = resistant, no inhibition; green = susceptible, high inhibition). *Ceramium* sp., and A*. armata* were excluded to aid visualization as they produced extremely high inhibition on the majority of the isolates. White cells in the figure indicate missing data. The phylogenetic tree was generated using a GTR model of nucleotide substitution and a GAMMA model of rate heterogeneity in RaxML

We obtained 26 high‐quality *S. aureus* genomic sequences, which revealed that a large proportion of known clinical *S. aureus* diversity was captured, with common sequence types (STs) from nosocomial infections (ST22; Holden et al., 2013) (ST250; Enright et al., 2002), community‐associated lineages (ST1; Earls et al., 2017), (ST59; Qu et al., 2014) and some strains more commonly associated with agricultural environments (ST5; Hau et al., 2018) (ST1245/CC130; Bortolami et al., 2017) represented within the collection. The antibiotic resistance profiles across the phylogenetic diversity of this *S. aureus* collection indicated a range of high and low antibiotic resistance isolates, in keeping with the broad population diversity captured within the collection (Fig. S3). Visualization of the susceptibility of *S. aureus* isolates to seaweed extracts alongside the phylogenetic tree of the *S. aureus* sample collection indicated limited genotype–phenotype clustering with large variation between seaweed extract treatments (Figure 7, red = resistant, green = susceptible).

## 4 DISCUSSION

We here, to our knowledge for the first time, demonstrate that bacterial resistance to clinical antibiotics is positively correlated with resistance to natural antimicrobials. A subset of seaweed extracts showed antimicrobial activity patterns similar to those of clinical antibiotics, a pattern that is consistent with cross‐resistance, where active compounds are structurally similar and/or target the same bacterial pathway (Baker‐Austin et al., 2006). The potential of cross‐resistance between natural antimicrobials and clinical antibiotics has important implications for human health. Recent, careful experimentation has demonstrated that ecologically relevant concentrations of secondary metabolites as exuded in the seaweed‐water boundary layer can select which bacteria can successfully settle (Lachnit, Fischer, Künzel, Baines, & Harder, 2013; Lachnit, Wahl, & Harder, 2009). Taken together, these findings suggest that seaweeds can select for colonizing bacteria that are resistant to their metabolites and that it is possible these same bacteria could also be more resistant to clinical antibiotics.

Many human pathogens can be found in environmental reservoirs, with *S. aureus* able to survive for significant periods in coastal waters (Levin‐Edens, Bonilla, Meschke, & Roberts, 2011; Tolba et al., 2008). Although the potential for prolonged (co‐)evolution of S*. aureus* in the marine environment might be limited, we note that humans could be exposed to a whole range of human pathogens, increasing the scope for resistance evolution and exposure. For instance, it has been estimated that there are over six million exposure events to cephalosporin‐resistant *E. coli* through recreational use of coastal bathing water in England and Wales alone (Leonard, Zhang, Balfour, Garside, & Gaze, 2015). Increased persistence of multidrug‐resistant pathogens settling on seaweeds in polluted coastal waters and possible in situ antibiotic resistance evolution of pathogens via mutation or the lateral transfer of resistance genes from bacteria native to seaweeds thus warrants further investigation. We also note that the potential for any “biotic co‐selection” extends beyond seaweeds and the wider marine environment and could potentially be mediated by different organisms and in terrestrial habitats.

Rather than using bacterial antibiotic‐resistant mutants generated through mutational processes in short‐term evolution experiments (e.g., Imamovic & Sommer, 2013), we used a genomically diverse set of pathogenic isolates more representative of resistance evolution to test for patterns of cross‐resistance. The antibiotic resistance profiles indicated a range of high and low resistance isolates, in keeping with the broad population diversity captured within the collection (Figure S3). No clear congruence could be observed between genomic relatedness and seaweed extract resistance. Some seaweed extracts showed generally low or high antimicrobial activity, but the extracts that showed variation in activity often did so across the entire phylogeny (e.g., see the activity of *C. melagonium*, *C. officinalis* and *Cladostephus spongiosus* in Figure 6). The uncoupling of phylogenetic relatedness and resistance patterns could have arisen due to a variety of reasons. The chemically diverse nature of extracts could mean a wide range of mechanisms underlies resistance phenotypes, from more targeted antibiotic‐like activity to unspecific biocidal activity. Lateral gene transfer is expected to unlink the carriage of genes involved in resistance and overall genomic relatedness. However, the fact that resistance is observed even within a clonal complex (e.g., ST22, Figure 6) makes divergence in resistance due to single point mutations likely as well.

Two extracts showed significantly less activity on strains that were more antibiotic resistant. The activity of other extracts showed relatively low variation in inhibition across the test panel, which in part could be caused by antimicrobial resistance mechanisms distinct from clinical resistance mechanisms. For instance, *A. armata* showed a particularly low level of variation in activity across the test panel; anti‐*S. aureus* activity of this species has been shown to be due to the production of bromoform and dibromoacetic acid (Paul, de Nys, & Steinberg, 2006). Brominated compounds act as mutagens (DeMarini, Perry, & Shelton, 1994; Kargalioglu, McMillan, Minear, & Plewa, 2002) which are not expected to act differently in strains with different antibiotic resistance. In a more direct comparison based on individual antibiotics and extracts, diverse patterns of cross‐resistance and collateral sensitivity could be observed, suggesting a wide diversity in modes of action and resistance mechanisms. Collateral sensitivity was observed between seaweed extracts and a range of antibiotics, including β‐lactams, fluoroquinolones and lincosamides.

Humankind has resorted to natural products to treat infections throughout history (Harrison et al., 2015), and the majority of drugs, including antibiotics, are natural products or have been derived from natural products (Butler & Buss, 2006; J. W.‐H. Li & Vederas, 2009). Decreases in profitability and biotechnological advances have meant that the search for novel pharmaceuticals has increasingly been led by high‐throughput screening of synthetic libraries (Clardy, Fischbach, & Walsh, 2006; J. W.‐H. Li & Vederas, 2009). Although modifications of existing structures with promising activity are relatively easy to generate and screen, concerns have been raised about the efficiency of this approach, as truly novel modes of actions are unlikely to be discovered (Chopra, 2012). In contrast, natural products represent a vastly richer biochemical diversity, which moreover is based on an evolutionary history that has optimized physiological function through natural selection (Clardy & Walsh, 2004). Considering that only a single bacterial target species was assayed, the prevalence and diversity of antimicrobial activity of seaweed extracts confirm the promise that natural antimicrobials hold.

We hope that correlational analyses such as employed here can be used to facilitate identification of novel extracts bearing promise for costly and time‐intensive downstream active compound identification and pharmacological testing. More generally, we contend that multidisciplinary approaches combining insights from microbiology, evolutionary ecology and ideally biochemistry are necessary to better understand both the potential of novel antimicrobials and the threats of antimicrobial resistance in the environment.

## CONFLICT OF INTEREST

None declared.

## Data Availability

Raw data have been deposited on Dryad https://doi.org/10.5061/dryad.86pg46n.
