# Supplementary material for: Patterns of cross‐resistance and collateral sensitivity between clinical antibiotics and natural antimicrobials
Source: Evol Appl. 2019 Jan 28;12(5):878–87. doi: 10.1111/eva.12762 (PMC6503891; doi:10.1111/eva.12762)
Supplement: Supplementary file 1 [file EVA-12-878-s001.pdf]

Supplementary Material "Cross-resistance and collateral sensitivity between clinical antibiotics and natural antimicrobials "

Contains two Supplementary Tables and three Supplementary Figures

**Table S1. Seaweed species used to generate 60% methanol extracts**					
Species(abv.)	Group	Collection date	Location	Coordinates	Anti-*S. aureus* activity
Ascophyllum nodosum (AN)	Phaeophyceae	03.09.14	Falmouth	50.148032, -5.054714	+++
*Asparagopsis armata (AA)**	Rhodophyceae	03.09.15	Falmouth	50.148032, -5.054714	+++
Chaetomorpha melagonium (CM)	Chlorophyceae	24.02.15	Penzance	50.114944,-5.531528	++
Ceramium rubrum (CR)	Rhodophyceae	07.02.15	Falmouth	50.148032, -5.054714	++
Ceramium sp. (CER)	Rhodophyceae	03.09.14	Falmouth	50.148032, -5.054714	+++
Champia parvula	Rhodophyceae	24.02.15	Penzance	50.114944,-5.531528	-
Chorda filum	Phaeophyceae	05.09.14	Pothleven	50.084042, -5.317190	-
Chondrus crispus	Rhodophyceae	24.02.15	Penzance	50.114944,-5.531528	-
*Chrysymenia wrightii**	Rhodophyceae	31.10.14	Flushing	50.163895, -5.070654	-
Cladophora rupestris (CLR)	Chlorophyceae	31.10.14	Flushing	50.163895, -5.070654	+
Cladostephus spongiosus (CS)	Phaeophyceae	03.09.14	Falmouth	50.148032, -5.054714	++
Colpomenia peregrina	Phaeophyceae	03.09.14	Falmouth	50.148032, -5.054714	-
Corallina officinalis (CO)	Rhodophyceae	03.09.14	Falmouth	50.148032, -5.054714	+
Cystoseria baccata (CB)	Phaeophyceae	03.09.14	Falmouth	50.148032, -5.054714	+++
Cystoseira nodicaulis (CN)	Phaeophyceae	03.09.14	Falmouth	50.148032, -5.054714	+++
Cystoseria tamariscifolia (CT)	Phaeophyceae	03.09.14	Falmouth	50.148032, -5.054714	+++
Dictyota dichotoma (DD)	Phaeophyceae	05.09.14	Falmouth	50.144793, -5.048631	+
Dilsea carnosa	Rhodophyceae	24.02.15	Penzance	50.114944,-5.531528	-
Desmarestia ligulata (DL)	Phaeophyceae	05.09.14	Porthleven	50.084042, -5.317190	+++
Dumontia contorta	Rhodophyceae	07.02.15	Falmouth	50.148032, -5.054714	-
Fucus serratus (FS)	Phaeophyceae	03.09.14	Falmouth	50.148032, -5.054714	+++
Fucus vesiculosus (FV)	Phaeophyceae	03.09.14	Falmouth	50.148032, -5.054714	+++
Furcellaria lumbricalis (FL)	Rhodophyceae	24.02.15	Penzance	50.114944,-5.531528	+++
Gelidium pulchella	Rhodophyceae	22.02.15	Falmouth	50.148032, -5.054714	-
*Grateloupia turuturu**	Rhodophyceae	24.02.15	Penzance	50.114944,-5.531528	-
Himanthalia elongata (HE)	Phaeophyceae	03.09.14	Falmouth	50.148032, -5.054714	+++

Halidrys siliquosa (HS)	Phaeophyceae	24.02.15	Penzance	50.114944,-5.531528	+++
Jania rubens (JR)	Rhodophyceae	24.02.15	Penzance	50.114944,-5.531528	+
Laminaria digitata (LD)	Phaeophyceae	03.09.14	Falmouth	50.148032, -5.054714	+++
Leathesia marina	Phaeophyceae	03.09.14	Falmouth	50.148032, -5.054714	-
Laminaria saccharina	Phaeophyceae	03.09.14	Falmouth	50.148032, -5.054714	-
Mastocarpus stellatus	Phaeophyceae	03.09.14	Falmouth	50.148032, -5.054714	-
Osmundea truncata	Rhodophyceae	22.02.15	Falmouth	50.148032, -5.054714	-
Palmaria palmata	Rhodophyceae	03.09.14	Falmouth	50.148032, -5.054714	-
Plumaria plumosa (PP)	Rhodophyceae	22.02.15	Falmouth	50.148032, -5.054714	++
Polyides rotundus	Rhodophyceae	03.09.14	Falmouth	50.148032, -5.054714	-
Polysiphonia elongata (PE)	Rhodophyceae	24.02.15	Penzance	50.114944,-5.531528	+++
Polysiphonia fucoides	Phaeophyceae	03.09.14	Falmouth	50.148032, -5.054714	-
Rhodomela confervoides (RC)	Rhodophyceae	24.02.15	Penzance	50.114944,-5.531528	+++
Saccorhiza polyschides	Phaeophyceae	22.02.15	Falmouth	50.148032, -5.054714	-
Sargassum muticum (SM)*	Phaeophyceae	03.09.14	Falmouth	50.148032, -5.054714	+++
*Soliera chordalis**	Rhodophyceae	22.02.15	Falmouth	50.148032, -5.054714	-
Sphaerococcus coronopifolius	Rhodophyceae	05.09.14	Falmouth	50.144793, -5.048631	-
Spyridia griffithsiana (SG)	Rhodophyceae	24.02.15	Penzance	50.114944,-5.531528	++
Stypocaulon scoparium	Phaeophyceae	24.02.15	Penzance	50.114944, -5.531528	-
Ulva intestinalis	Chlorophyceae	03.09.14	Falmouth	50.148032, -5.054714	-
Ulva lactuca (UL)	Chlorophyceae	03.09.14	Falmouth	50.148032, -5.054714	++
*Undaria pinnatifida (UP)**	Phaeophyceae	07.02.15	Falmouth	50.163122, -5.083751	+++

Inhibition of *S. aureus* strains (n=28) was scored as: 27 (+++), >14 (++), >0<14 (+), 0 (-). *= invasive species

Table S2: VITEK clinical antibiotics minimal inhibitory concentration (MIC) data for all 28 *Staphylococcus aureus* isolates

Strain	BP	OXA	CEF	AMI	GEN	TOB	CIP	LEV	OFL	CLA	ERY	CLI	LIN	DAP	TEI	VAN	TET	FUS	MUP	CHL	RIF	TRI
4244	**>=0.5***	**>=4***	*		<=0.5		**>=8***	*	*	*	**>=8***	**>=4***	2	0.5	<=0.5	1	**>=16***	<=0.5	<=2	8	<=0.03	<=0.5
3887	**>=0.5***	**>=4***	*	*	**>=16***	*	**>=8***	*	*		0.5	0.25	2	0.25	<=0.5	1	<=1	<=0.5	<=2	<=4	<=0.03	1
2254	**>=0.5***	**>=4***	*		<=0.5		**>=8***	*	*	*	**>=8***	0.25	2	0.25	<=0.5	1	<=1	<=0.5	<=2	<=4	<=0.03	<=0.5
3865	**>=0.5***	**>=4***	*		<=0.5		**>=8***	*	*	*	**>=8***	**0.25***	2	0.25	<=0.5	1	<=1	<=0.5	<=2	8	<=0.03	<=0.5
3344	**>=0.5***	**>=4***	*		<=0.5		2	*	*	*	**>=8***	**0.25***	2	0.25	<=0.5	1	<=1	<=0.5	<=2	<=4	<=0.03	<=0.5
2522	**>=0.5***	**>=4***	*		<=0.5		**>=8***	*	*	*	**>=8***	**0.25***	2	0.5	<=0.5	1	<=1	<=0.5	<=2	8	<=0.03	<=0.5
3349	**>=0.5***	0.5			<=0.5		**>=8***	*	*	*	**>=8***	0.25	2	0.5	<=0.5	1	<=1	<=0.5	<=2	<=4	<=0.03	**>=16**
2458	**>=0.5***	**>=4***	*		<=0.5		<=0.5			*	**>=8***	**0.25***	2	0.25	1	1	<=1	**16***	<=2	8	<=0.03	1
2564	**>=0.5***	**>=4***	*		<=0.5		**>=8***	*	*		1	0.25	2	0.25	<=0.6	<=0.5	<=1	**8***	<=2	8	<=0.03	<=0.5
3935	**>=0.5***	**>=4***	*		<=0.5		**>=8***	*	*		1	0.25	2	0.25	<=0.5	1	<=1	**>=32**	<=2	8	<=0.03	<=0.5
2343	**>=0.5***	**>=4***	*		<=0.5		**>=8***	*	*		1	0.25	2	0.25	<=0.5	<=0.5	<=1	<=0.5	<=2	8	<=0.03	<=0.5
2636	**>=0.5***	0.5			<=0.5		1**	**	**	*	**2***	0.25	2	0.5	<=0.5	1	<=1	<=0.5	<=2	8	<=0.03	<=0.5
3484	**>=0.5***	0.5			<=0.5		<=0.5			*	**>=8***	**0.25***	2	0.25	<=0.5	1	<=1	**>=32***	<=2	<=4	<=0.03	1
2277	**>=0.5***	**>=4***	*		<=0.5		<=0.5				0.5	0.25	2	<=0.12	<=0.5	1	<=1	**>=32***	<=2	8	<=0.03	1
3826	**>=0.5***	**>=4***	*		<=0.5		<=0.5				0.5	0.25	4	0.25	**4***	1	<=1	<=0.5	<=2	8	<=0.03	<=0.5
2967	**>=0.5***	**>=4***	*		<=0.5		<=0.5				1	0.25	2	0.25	<=0.5	1	<=1	<=0.5	<=2	8	<=0.03	<=0.5
3526	**>=0.5***	0.5			<=0.5		<=0.5			*	**>=8***	0.25	2	<=0.12	<=0.5	1	<=1	<=0.5	<=2	<=4	<=0.03	<=0.5
3379	**>=0.5***	<=0.25			<=0.5		<=0.5			*	**2***	0.25	2	0.25	<=0.5	1	<=1	<=0.5	<=2	<=4	<=0.03	<=0.5
2424	**>=0.5***	**>=4***	*		<=0.5		<=0.5				0.5	0.25	2	0.25	1	1	<=1	<=0.5	<=2	8	<=0.03	<=0.5
3737	**>=0.5***	0.5			<=0.5		<=0.5				1	0.25	2	0.25	<=0.5	1	**>=16***	<=0.5	<=2	8	<=0.03	<=0.5
3253	**>=0.5***	<=0.25			<=0.5		<=0.5				1	<=0.12	2	0.25	<=0.5	<=0.5	<=1	<=0.5	<=2	<=4	<=0.03	<=0.5
2882	**>=0.5***	<=0.25			<=0.5		<=0.5				1	0.25	2	0.25	<=0.5	1	<=1	<=0.5	<=2	<=4	<=0.03	<=0.5
2369	**>=0.5***	<0.25			<=0.5		<=0.5				0.5	0.25	4	0.25	<=0.5	1	<=1	1	<=2	8	<=0.03	<=0.5
3729	**>=0.5***	0.5			<=0.5		<=0.5				1	0.25	2	0.25	<=0.5	1	<=1	<=0.5	<=2	<=4	<=0.03	<=0.5
2832	**>=0.5***	<=0.25			<=0.5		<=0.5				1	0.25	2	0.5	<=0.5	1	<=1	<=0.5	<=2	<=4	<=0.03	<=0.5
3228	**>=0.5***	<=0.25			<=0.5		<=0.5				1	0.25	2	1	<=0.5	1	<=1	<=0.5	<=2	<=4	<=0.03	1
3565	0.12	<=0.25			<=0.5		<=0.5				1	0.25	2	0.25	<=0.5	1	<=1	<=0.5	<=2	<=4	<=0.03	<=0.5
2934	0.06	<=0.25			<=0.5		<=0.5				0.5	0.25	2	0.5	<=0.5	<=0.5	<=1	<=0.5	<=2	<=4	<=0.03	<=0.5

BP: Benzylpenicillin, OXA: Oxacillin, CEF: Ceftriaxone, AMI: Amikacin, GEN: Gentamicin, TOB: Tobramycin, CIP: Ciprofloxacin, LEV: Levofloxacin, OFL: Ofloxacin, CLA: Clarithromycin, ERY: Erythromycin, CLI: Clindamycin, LIN: Linezolid, DAP: Daptomycin, TEI: Teicoplanin, VAN: Vancomycin, TET: Tetracycline, FUS: Fusidic acid, MUP: Mupirocin, CHL: Chloramphenicol, RIF: Rifampicin, TRI: Trimethoprim. Breakpoints expressed in mg/L. * = Resistant MIC, ** = Intermediate MIC.

Figure S1: An example of a Kirby-Bauer disc diffusion assay with positive control disc (Imipenem, 4 mg/l, 'IMP') and negative control (60% methanol, 'M'). Some of the discs labelled 1-14 show inhibition of *S. aureus* growth due to the diffusion of extracts into the agar.

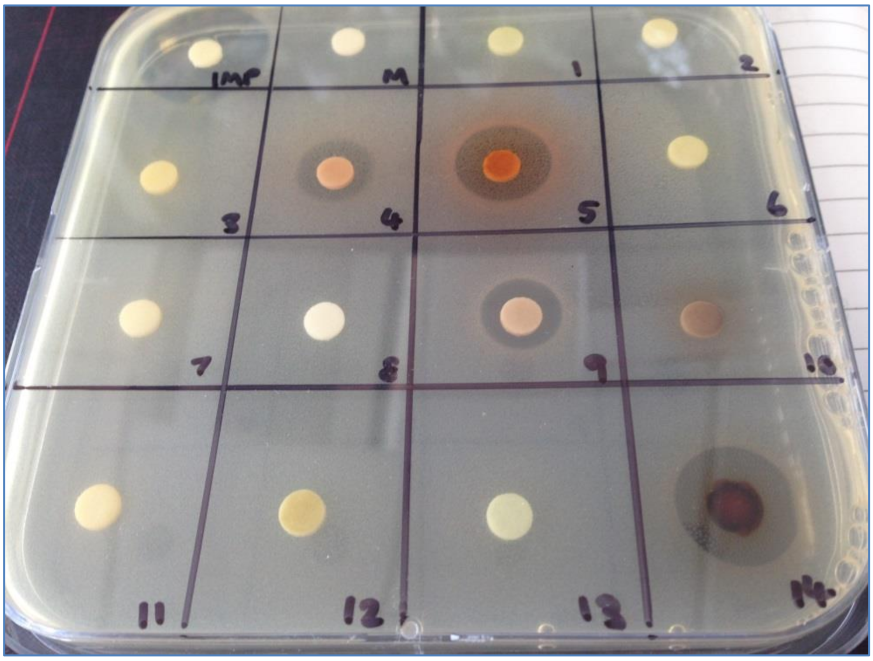

Figure S2. Inhibition zones of *Cystoseira baccata* (CB), *C. nodicaulis* (CN) and *C. tamariscifolia* (CT) on *E. coli* K12 (EC), *Klebsiella pneumonia* (clinical isolate 14U080593-1 Royal Cornwall Hospital) (KP) and *Pseudomonas aeruginosa* PAO1 (PA).

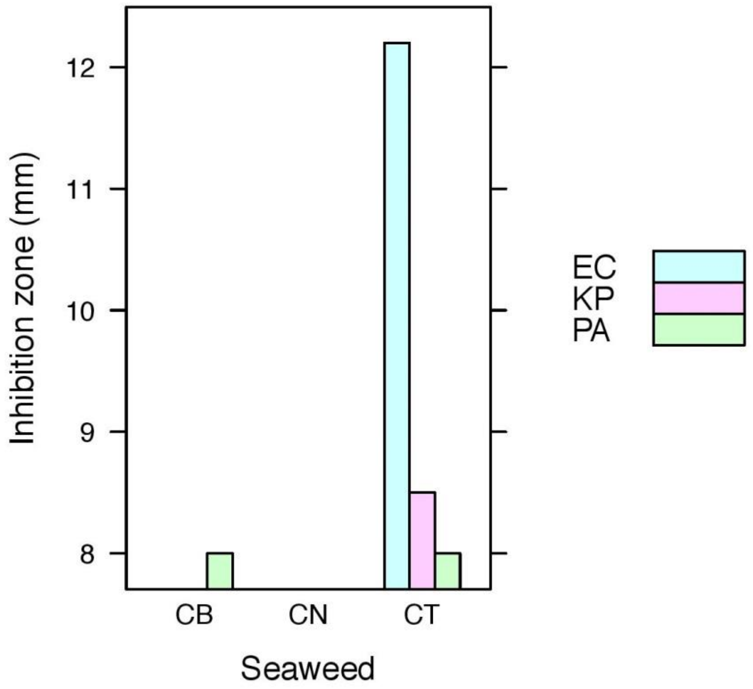

Figure S3. A maximum-likelihood phylogenetic tree based on whole genome sequence data of 26 *S. aureus* genomes mapped to EMRSA15 reference genome HO 5096 0412 (2,832,299 bp). The panel on the right indicates susceptibility to 22 antibiotics assayed using the VITEK system with resistance/sensitivity assigned using designated MIC breakpoints for each antibiotic (red = resistant, white = susceptible). The phylogenetic tree was generated using a GTR model of nucleotide substitution and a GAMMA model of rate heterogeneity in RaxML.

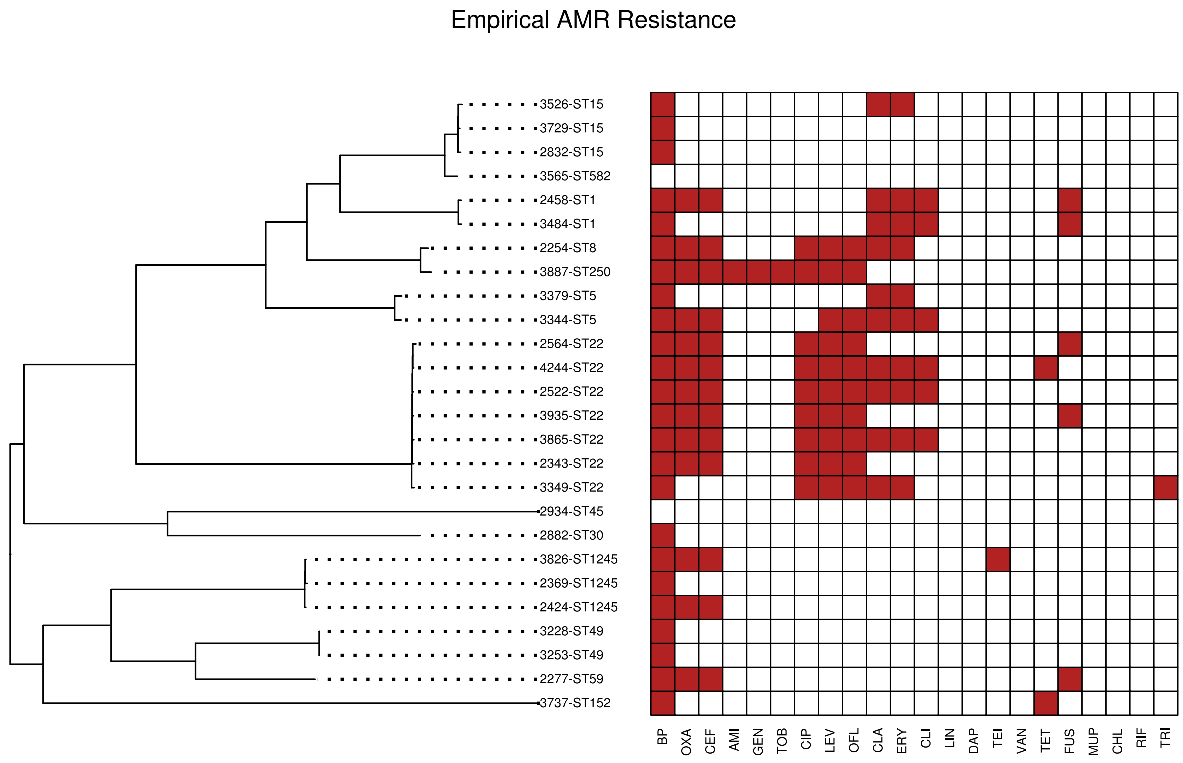

BP: Benzylpenicillin, OXA: Oxacillin, CEF: Ceftriaxone, AMI: Amikacin, GEN: Gentamicin, TOB: Tobramycin, CIP: Ciprofloxacin, LEV: Levofloxacin, OFL: Ofloxacin, CLA: Clarithromycin, ERY: Erythromycin, CLI:Clindamycin, LIN: Linezolid, DAP: Daptomycin, TEI: Teicoplanin, VAN: Vancomycin, TET: Tetracycline, FUS: Fusidic acid, MUP: Mupirocin, CHL: Chloramphenicol, RIF: Rifampicin, TRI: Trimethoprim.
